# 2,4-Bis(2-bromo­phen­yl)-3-aza­bicyclo­[3.3.1]nonan-9-one

**DOI:** 10.1107/S1600536808037501

**Published:** 2008-11-20

**Authors:** P. Parthiban, V. Ramkumar, Min Sung Kim, Se Mo Son, Yeon Tae Jeong

**Affiliations:** a Division of Image Science and Information Engineering, Pukyong National University, Busan 608 739, Republic of Korea; b Department of Chemistry, IIT Madras, Chennai, Tamilnadu, India

## Abstract

In the mol­ecular structure of the title compound, C_20_H_19_Br_2_NO, the fused six-membered heterocyclic and cyclo­hexane rings adopt a twin-chair conformation with equatorial orientations of all the substituents. Both the *ortho*-bromo substituents of the benzene rings are oriented towards the carbonyl group; the dihedral angle between the ring planes is 29.13 (3)°. In the crystal structure, the N—H group does not participate in any hydrogen bonds.

## Related literature

For 3-aza­bicyclo­nonan-9-ones and their significance as bio-active mol­ecules, see: Barker *et al.* (2005 ▶); Jeyaraman & Avila (1981 ▶). For puckering parameters, see: Cremer & Pople (1975 ▶); Web & Becker (1967 ▶). For a similiar structure see; Parthiban *et al.* (2008 ▶).
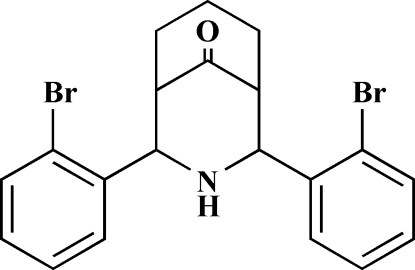


## Experimental

### 

#### Crystal data

C_20_H_19_Br_2_NO
*M* *_r_* = 449.18
Triclinic, 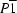

*a* = 7.8389 (3) Å
*b* = 10.5770 (3) Å
*c* = 11.0274 (3) Å
α = 101.099 (2)°
β = 93.725 (2)°
γ = 97.399 (1)°
*V* = 885.94 (5) Å^3^
*Z* = 2
Mo *K*α radiation
μ = 4.58 mm^−1^
*T* = 298 (2) K
0.45 × 0.38 × 0.35 mm

#### Data collection

Bruker APEXII CCD diffractometer
Absorption correction: multi-scan (*SADABS*; Bruker, 1999 ▶) *T*_min_ = 0.232, *T*_max_ = 0.297 (expected range = 0.157–0.201)
10959 measured reflections
4098 independent reflections
3266 reflections with *I* > 2σ(*I*)
*R*_int_ = 0.017

#### Refinement

*R*[*F* ^2^ > 2σ(*F* ^2^)] = 0.027
*wR*(*F* ^2^) = 0.060
*S* = 1.00
4098 reflections
221 parameters
H atoms treated by a mixture of independent and constrained refinement
Δρ_max_ = 0.58 e Å^−3^
Δρ_min_ = −0.56 e Å^−3^

### 

Data collection: *APEX2* (Bruker, 2004 ▶); cell refinement: *SAINT-Plus* (Bruker, 2004 ▶); data reduction: *SAINT-Plus*; program(s) used to solve structure: *SHELXS97* (Sheldrick, 2008 ▶); program(s) used to refine structure: *SHELXL97* (Sheldrick, 2008 ▶); molecular graphics: *ORTEP-3* (Farrugia, 1997 ▶); software used to prepare material for publication: *SHELXL97*.

## Supplementary Material

Crystal structure: contains datablocks global, I. DOI: 10.1107/S1600536808037501/hb2843sup1.cif

Structure factors: contains datablocks I. DOI: 10.1107/S1600536808037501/hb2843Isup2.hkl

Additional supplementary materials:  crystallographic information; 3D view; checkCIF report

## Figures and Tables

**Fig. 1.**
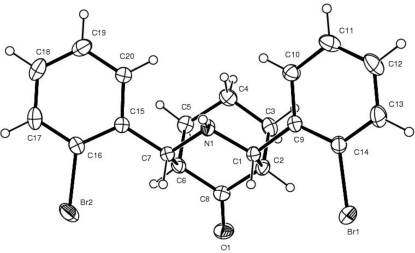
The molecular structure of (I) with non-hydrogen atoms represented as 30% probability ellipsoids.

## supplementary crystallographic information

### Comment

3-Azabicyclononan-9-ones are important class of compounds due to their significance as bio-active molecules (Jeyaraman & Avila, 1981; Barker et al., 2005).

The title compound, (I), exists in a chair–chair conformation with equatorial orientations of the ortho bromo-phenyl groups on each side of the secondary amino group with the torsion angles of C8—C2—C1—C9 and C8—C6—C7—C15 being 177.88 (4) and 179.42 (6)°, respectively. In both aryl groups, the bromo substituents point towards the carbonyl group and the dihedral angle between the ring planes is 29.13 (3)°. The piperidine ring adopts near ideal chair conformation with the deviation of ring atoms N1 and C8 from the C1/C2/C6/C7 plane by -0.635 (3)and 0.705 (3)Å, respectively, Q_T_ = 0.599 (2)Å, q(2)=0.047 (2)Å, q(3)=0.597 (2)Å, θ = 4.71 (19)° Cremer & Pople, 1975; Web & Becker, 1967), whereas the cyclohexane ring atoms C4 and C8 deviate from the C2/C3/C5/C6 plane by -0.539 (4) and 0.725 (3)Å, respectively, Q_T_ = 0.565 (2)Å, q(2)=0.141 (2)Å, q(3)=0.548 (2)Å, θ = 14.4 (2)°, indicating a deviation from the ideal chair conformation of the cyclohexane part in the title compound. The crystal structure is stabilized by the intermolecular van der Waals interactions.

### Experimental

A mixture of cyclohexanone (0.05 mol) and *ortho* bromobenzaldehyde (0.1 mol) was added to a warm solution of ammonium acetate (0.075 mol) in 50 ml of absolute ethanol. The mixture was gently warmed on a hot plate until a yellow colour was formed and then cooled to room temperature. Then, 50 ml of ether was added and allowed to stir over night at room temperature. At the end, the crude azabicyclic ketone was separated by filtration and washed with 1:5 v/v ethanol–ether mixture till the solid became colourless. Recrystallization of the compound from acetone gave colourless blocks of (I).

### Refinement

The nitrogen-bound H atom was located in a difference map and refined isotropically. The other hydrogen atoms were fixed geometrically (C—H = 0.93–0.98Å) and refined as riding with U_iso_(H) = 1.2U_eq_(C).

### Crystal data

**Table:**

C_20_H_19_Br_2_NO	*Z* = 2
*M**_r_* = 449.18	*F*_000_ = 448
Triclinic, *P*1	*D*_x_ = 1.684 Mg m^−^^3^
Hall symbol: -P 1	Mo *K*α radiation λ = 0.71073 Å
*a* = 7.8389 (3) Å	Cell parameters from 5642 reflections
*b* = 10.5770 (3) Å	θ = 2.5–28.2º
*c* = 11.0274 (3) Å	µ = 4.58 mm^−^^1^
α = 101.099 (2)º	*T* = 298 (2) K
β = 93.725 (2)º	Block, colourless
γ = 97.399 (1)º	0.45 × 0.38 × 0.35 mm
*V* = 885.94 (5) Å^3^	

### Data collection

**Table:**

Bruker APEXII CCD diffractometer	4098 independent reflections
Radiation source: fine-focus sealed tube	3266 reflections with *I* > 2σ(*I*)
Monochromator: graphite	*R*_int_ = 0.017
*T* = 298(2) K	θ_max_ = 28.3º
ω scans	θ_min_ = 1.9º
Absorption correction: Multi-scan(SADABS; Bruker, 1999)	*h* = −10→10
*T*_min_ = 0.232, *T*_max_ = 0.297	*k* = −13→13
10959 measured reflections	*l* = −14→14

### Refinement

**Table:**

Refinement on *F*^2^	Secondary atom site location: difference Fourier map
Least-squares matrix: full	Hydrogen site location: inferred from neighbouring sites
*R*[*F*^2^ > 2σ(*F*^2^)] = 0.027	H atoms treated by a mixture of independent and constrained refinement
*wR*(*F*^2^) = 0.060	*w* = 1/[σ^2^(*F*_o_^2^) + (0.0207*P*)^2^ + 0.5638*P*] where *P* = (*F*_o_^2^ + 2*F*_c_^2^)/3
*S* = 1.00	(Δ/σ)_max_ = 0.002
4098 reflections	Δρ_max_ = 0.58 e Å^−^^3^
221 parameters	Δρ_min_ = −0.56 e Å^−^^3^
Primary atom site location: structure-invariant direct methods	Extinction correction: none

### Special details

**Table:**

Geometry. All e.s.d.'s (except the e.s.d. in the dihedral angle between two l.s. planes)are estimated using the full covariance matrix. The cell e.s.d.'s are takeninto account individually in the estimation of e.s.d.'s in distances, anglesand torsion angles; correlations between e.s.d.'s in cell parameters are onlyused when they are defined by crystal symmetry. An approximate (isotropic)treatment of cell e.s.d.'s is used for estimating e.s.d.'s involving l.s. planes.
Refinement. Refinement of *F*^2^ against ALL reflections. The weighted *R*-factor *wR* andgoodness of fit *S* are based on *F*^2^, conventional *R*-factors *R* are basedon *F*, with *F* set to zero for negative *F*^2^. The threshold expression of*F*^2^ > σ(*F*^2^) is used only for calculating *R*-factors(gt) *etc*. and isnot relevant to the choice of reflections for refinement. *R*-factors basedon *F*^2^ are statistically about twice as large as those based on *F*, and *R*-factors based on ALL data will be even larger.

### Fractional atomic coordinates and isotropic or equivalent isotropic displacement parameters (Å^2^)

**Table:**

	*x*	*y*	*z*	*U*_iso_*/*U*_eq_	
Br1	−0.00438 (3)	0.47719 (2)	0.77681 (2)	0.05065 (8)	
Br2	0.16949 (3)	1.23193 (3)	1.01448 (2)	0.06070 (9)	
C1	0.2124 (2)	0.74756 (18)	0.75331 (18)	0.0301 (4)	
H1	0.1437	0.7370	0.8228	0.036*	
C2	0.0889 (2)	0.76537 (19)	0.64395 (19)	0.0344 (4)	
H2	−0.0049	0.6918	0.6254	0.041*	
C3	0.1735 (3)	0.7773 (2)	0.5238 (2)	0.0428 (5)	
H3A	0.2294	0.7011	0.4980	0.051*	
H3B	0.0839	0.7778	0.4591	0.051*	
C4	0.3064 (3)	0.8987 (2)	0.5360 (2)	0.0432 (5)	
H4A	0.4119	0.8865	0.5806	0.052*	
H4B	0.3333	0.9102	0.4539	0.052*	
C5	0.2434 (3)	1.0214 (2)	0.60396 (19)	0.0392 (5)	
H5A	0.1647	1.0500	0.5468	0.047*	
H5B	0.3418	1.0895	0.6280	0.047*	
C6	0.1515 (2)	1.00528 (19)	0.72040 (18)	0.0331 (4)	
H6	0.0967	1.0828	0.7479	0.040*	
C7	0.2692 (2)	0.98341 (18)	0.83118 (17)	0.0294 (4)	
H7	0.1987	0.9747	0.9001	0.035*	
C8	0.0132 (2)	0.8886 (2)	0.68529 (18)	0.0342 (4)	
C9	0.3014 (2)	0.62834 (18)	0.72007 (17)	0.0304 (4)	
C10	0.4692 (3)	0.6383 (2)	0.6847 (2)	0.0389 (5)	
H10	0.5265	0.7194	0.6796	0.047*	
C11	0.5525 (3)	0.5302 (2)	0.6570 (2)	0.0495 (6)	
H11	0.6641	0.5392	0.6328	0.059*	
C12	0.4711 (3)	0.4095 (2)	0.6651 (2)	0.0556 (6)	
H12	0.5280	0.3371	0.6472	0.067*	
C13	0.3055 (3)	0.3958 (2)	0.6995 (2)	0.0482 (6)	
H13	0.2496	0.3142	0.7047	0.058*	
C14	0.2228 (3)	0.50413 (19)	0.72632 (19)	0.0350 (4)	
C15	0.4125 (2)	1.09742 (18)	0.87430 (17)	0.0290 (4)	
C16	0.3860 (3)	1.21258 (19)	0.95107 (18)	0.0337 (4)	
C17	0.5159 (3)	1.3181 (2)	0.9863 (2)	0.0436 (5)	
H17	0.4946	1.3938	1.0380	0.052*	
C18	0.6757 (3)	1.3104 (2)	0.9446 (2)	0.0499 (6)	
H18	0.7629	1.3812	0.9670	0.060*	
C19	0.7071 (3)	1.1976 (2)	0.8696 (2)	0.0482 (6)	
H19	0.8161	1.1920	0.8421	0.058*	
C20	0.5772 (3)	1.0923 (2)	0.8347 (2)	0.0381 (5)	
H20	0.6003	1.0166	0.7839	0.046*	
N1	0.3422 (2)	0.86343 (15)	0.79409 (16)	0.0304 (4)	
O1	−0.13950 (19)	0.89419 (17)	0.68681 (16)	0.0521 (4)	
H1A	0.407 (3)	0.851 (2)	0.850 (2)	0.040 (7)*	

### Atomic displacement parameters (Å^2^)

**Table:**

	*U*^11^	*U*^22^	*U*^33^	*U*^12^	*U*^13^	*U*^23^
Br1	0.04366 (13)	0.03775 (13)	0.06740 (17)	−0.00469 (9)	0.01281 (11)	0.00737 (11)
Br2	0.05389 (15)	0.06429 (18)	0.05949 (17)	0.02181 (12)	0.01497 (12)	−0.01039 (13)
C1	0.0296 (9)	0.0256 (10)	0.0349 (10)	0.0042 (7)	0.0038 (8)	0.0056 (8)
C2	0.0286 (9)	0.0300 (10)	0.0415 (11)	0.0019 (8)	−0.0038 (8)	0.0035 (9)
C3	0.0511 (13)	0.0398 (12)	0.0351 (11)	0.0125 (10)	−0.0022 (10)	−0.0001 (9)
C4	0.0505 (13)	0.0492 (13)	0.0325 (11)	0.0104 (10)	0.0097 (9)	0.0107 (10)
C5	0.0441 (11)	0.0369 (12)	0.0386 (12)	0.0063 (9)	−0.0008 (9)	0.0138 (9)
C6	0.0326 (10)	0.0299 (10)	0.0371 (11)	0.0102 (8)	0.0012 (8)	0.0046 (8)
C7	0.0300 (9)	0.0276 (10)	0.0302 (10)	0.0044 (7)	0.0033 (7)	0.0049 (8)
C8	0.0310 (9)	0.0405 (12)	0.0327 (11)	0.0088 (8)	−0.0003 (8)	0.0100 (9)
C9	0.0335 (9)	0.0278 (10)	0.0295 (10)	0.0060 (8)	0.0003 (8)	0.0046 (8)
C10	0.0357 (10)	0.0380 (12)	0.0448 (12)	0.0081 (9)	0.0059 (9)	0.0104 (9)
C11	0.0417 (12)	0.0553 (15)	0.0575 (15)	0.0220 (11)	0.0123 (11)	0.0135 (12)
C12	0.0657 (16)	0.0446 (14)	0.0637 (16)	0.0311 (12)	0.0140 (13)	0.0109 (12)
C13	0.0608 (15)	0.0297 (12)	0.0551 (14)	0.0101 (10)	0.0065 (11)	0.0083 (10)
C14	0.0371 (10)	0.0304 (10)	0.0362 (11)	0.0041 (8)	0.0025 (8)	0.0038 (8)
C15	0.0314 (9)	0.0280 (10)	0.0284 (10)	0.0053 (8)	0.0005 (7)	0.0079 (8)
C16	0.0403 (10)	0.0322 (11)	0.0297 (10)	0.0100 (8)	0.0010 (8)	0.0062 (8)
C17	0.0646 (15)	0.0278 (11)	0.0353 (12)	0.0034 (10)	−0.0054 (10)	0.0047 (9)
C18	0.0547 (14)	0.0427 (13)	0.0455 (13)	−0.0154 (11)	−0.0072 (11)	0.0108 (11)
C19	0.0346 (11)	0.0573 (15)	0.0500 (14)	−0.0043 (10)	0.0028 (10)	0.0117 (12)
C20	0.0337 (10)	0.0375 (11)	0.0411 (12)	0.0048 (9)	0.0047 (9)	0.0029 (9)
N1	0.0292 (8)	0.0249 (8)	0.0362 (9)	0.0055 (6)	−0.0050 (7)	0.0053 (7)
O1	0.0292 (7)	0.0588 (11)	0.0693 (11)	0.0119 (7)	0.0028 (7)	0.0120 (9)

### Geometric parameters (Å, °)

**Table:**

Br1—C14	1.903 (2)	C7—H7	0.9800
Br2—C16	1.897 (2)	C8—O1	1.207 (2)
C1—N1	1.465 (2)	C9—C10	1.393 (3)
C1—C9	1.515 (3)	C9—C14	1.394 (3)
C1—C2	1.552 (3)	C10—C11	1.382 (3)
C1—H1	0.9800	C10—H10	0.9300
C2—C8	1.505 (3)	C11—C12	1.373 (4)
C2—C3	1.539 (3)	C11—H11	0.9300
C2—H2	0.9800	C12—C13	1.373 (3)
C3—C4	1.524 (3)	C12—H12	0.9300
C3—H3A	0.9700	C13—C14	1.381 (3)
C3—H3B	0.9700	C13—H13	0.9300
C4—C5	1.525 (3)	C15—C20	1.393 (3)
C4—H4A	0.9700	C15—C16	1.391 (3)
C4—H4B	0.9700	C16—C17	1.387 (3)
C5—C6	1.539 (3)	C17—C18	1.369 (3)
C5—H5A	0.9700	C17—H17	0.9300
C5—H5B	0.9700	C18—C19	1.375 (4)
C6—C8	1.506 (3)	C18—H18	0.9300
C6—C7	1.554 (3)	C19—C20	1.385 (3)
C6—H6	0.9800	C19—H19	0.9300
C7—N1	1.457 (2)	C20—H20	0.9300
C7—C15	1.518 (2)	N1—H1A	0.81 (2)
			
N1—C1—C9	109.63 (15)	O1—C8—C2	124.48 (19)
N1—C1—C2	110.38 (16)	O1—C8—C6	123.98 (19)
C9—C1—C2	112.32 (16)	C2—C8—C6	111.51 (16)
N1—C1—H1	108.1	C10—C9—C14	116.51 (18)
C9—C1—H1	108.1	C10—C9—C1	121.26 (17)
C2—C1—H1	108.1	C14—C9—C1	122.21 (17)
C8—C2—C3	107.21 (17)	C11—C10—C9	121.5 (2)
C8—C2—C1	107.83 (16)	C11—C10—H10	119.3
C3—C2—C1	115.42 (16)	C9—C10—H10	119.3
C8—C2—H2	108.7	C12—C11—C10	120.3 (2)
C3—C2—H2	108.7	C12—C11—H11	119.9
C1—C2—H2	108.7	C10—C11—H11	119.9
C4—C3—C2	114.03 (17)	C13—C12—C11	119.9 (2)
C4—C3—H3A	108.7	C13—C12—H12	120.1
C2—C3—H3A	108.7	C11—C12—H12	120.1
C4—C3—H3B	108.7	C12—C13—C14	119.5 (2)
C2—C3—H3B	108.7	C12—C13—H13	120.3
H3A—C3—H3B	107.6	C14—C13—H13	120.3
C5—C4—C3	112.67 (18)	C13—C14—C9	122.3 (2)
C5—C4—H4A	109.1	C13—C14—Br1	116.76 (16)
C3—C4—H4A	109.1	C9—C14—Br1	120.89 (15)
C5—C4—H4B	109.1	C20—C15—C16	116.80 (18)
C3—C4—H4B	109.1	C20—C15—C7	120.81 (17)
H4A—C4—H4B	107.8	C16—C15—C7	122.36 (17)
C4—C5—C6	114.82 (17)	C17—C16—C15	122.0 (2)
C4—C5—H5A	108.6	C17—C16—Br2	116.64 (16)
C6—C5—H5A	108.6	C15—C16—Br2	121.32 (15)
C4—C5—H5B	108.6	C18—C17—C16	119.7 (2)
C6—C5—H5B	108.6	C18—C17—H17	120.2
H5A—C5—H5B	107.5	C16—C17—H17	120.2
C8—C6—C5	108.13 (16)	C17—C18—C19	119.9 (2)
C8—C6—C7	107.18 (16)	C17—C18—H18	120.0
C5—C6—C7	115.25 (16)	C19—C18—H18	120.0
C8—C6—H6	108.7	C18—C19—C20	120.3 (2)
C5—C6—H6	108.7	C18—C19—H19	119.9
C7—C6—H6	108.7	C20—C19—H19	119.9
N1—C7—C15	110.21 (15)	C19—C20—C15	121.3 (2)
N1—C7—C6	109.31 (15)	C19—C20—H20	119.3
C15—C7—C6	111.09 (15)	C15—C20—H20	119.3
N1—C7—H7	108.7	C7—N1—C1	113.89 (15)
C15—C7—H7	108.7	C7—N1—H1A	111.0 (16)
C6—C7—H7	108.7	C1—N1—H1A	108.6 (16)
			
N1—C1—C2—C8	−55.2 (2)	C9—C10—C11—C12	−0.6 (4)
C9—C1—C2—C8	−177.89 (16)	C10—C11—C12—C13	0.7 (4)
N1—C1—C2—C3	64.6 (2)	C11—C12—C13—C14	−0.3 (4)
C9—C1—C2—C3	−58.1 (2)	C12—C13—C14—C9	−0.2 (3)
C8—C2—C3—C4	55.1 (2)	C12—C13—C14—Br1	−178.65 (19)
C1—C2—C3—C4	−65.0 (2)	C10—C9—C14—C13	0.2 (3)
C2—C3—C4—C5	−45.4 (3)	C1—C9—C14—C13	−178.10 (19)
C3—C4—C5—C6	43.4 (3)	C10—C9—C14—Br1	178.65 (15)
C4—C5—C6—C8	−51.3 (2)	C1—C9—C14—Br1	0.3 (3)
C4—C5—C6—C7	68.6 (2)	N1—C7—C15—C20	23.6 (2)
C8—C6—C7—N1	58.74 (19)	C6—C7—C15—C20	−97.7 (2)
C5—C6—C7—N1	−61.6 (2)	N1—C7—C15—C16	−158.48 (18)
C8—C6—C7—C15	−179.43 (15)	C6—C7—C15—C16	80.2 (2)
C5—C6—C7—C15	60.2 (2)	C20—C15—C16—C17	0.5 (3)
C3—C2—C8—O1	113.0 (2)	C7—C15—C16—C17	−177.53 (18)
C1—C2—C8—O1	−122.1 (2)	C20—C15—C16—Br2	−178.76 (15)
C3—C2—C8—C6	−64.9 (2)	C7—C15—C16—Br2	3.2 (3)
C1—C2—C8—C6	59.9 (2)	C15—C16—C17—C18	0.3 (3)
C5—C6—C8—O1	−114.9 (2)	Br2—C16—C17—C18	179.52 (17)
C7—C6—C8—O1	120.3 (2)	C16—C17—C18—C19	−0.9 (3)
C5—C6—C8—C2	63.1 (2)	C17—C18—C19—C20	0.8 (4)
C7—C6—C8—C2	−61.7 (2)	C18—C19—C20—C15	0.0 (3)
N1—C1—C9—C10	−24.4 (3)	C16—C15—C20—C19	−0.6 (3)
C2—C1—C9—C10	98.7 (2)	C7—C15—C20—C19	177.4 (2)
N1—C1—C9—C14	153.82 (18)	C15—C7—N1—C1	178.86 (15)
C2—C1—C9—C14	−83.1 (2)	C6—C7—N1—C1	−58.8 (2)
C14—C9—C10—C11	0.2 (3)	C9—C1—N1—C7	−178.59 (16)
C1—C9—C10—C11	178.5 (2)	C2—C1—N1—C7	57.2 (2)
